# Effects on Gastroesophageal Reflux of Donkey Milk-Derived Human Milk Fortifier Versus Standard Fortifier in Preterm Newborns: Additional Data from the FortiLat Study

**DOI:** 10.3390/nu12072142

**Published:** 2020-07-18

**Authors:** Francesco Cresi, Elena Maggiora, Alice Pirra, Paola Tonetto, Carlotta Rubino, Laura Cavallarin, Marzia Giribaldi, Guido E. Moro, Chiara Peila, Alessandra Coscia

**Affiliations:** 1 Neonatal Care Unit, Sant’Anna Hospital, Città della Salute e della Scienza, Università degli Studi di Torino, 10126 Turin, Italy; francesco.cresi@unito.it (F.C.); alice.pirra@gmail.com (A.P.); paola.tonetto@unito.it (P.T.); carlotta.rubino@edu.unito.it (C.R.); chiara.peila@unito.it (C.P.); alessandra.coscia@unito.it (A.C.); 2 Institute of Sciences of Food Production, Italian National Research Council, 10126 Turin, Italy; laura.cavallarin@ispa.cnr.it; 3 Research Centre for Engineering and Agro-Food Processing, Council for Agricultural Research and Economics (CREA), 10100 Turin, Italy; marzia.giribaldi@ispa.cnr.it; 4 Italian Association of Human Milk Banks, Italy; guidoemoro@tiscali.it

**Keywords:** VLBW, GORD, very preterm, feeding intolerance, tolerance, cardiorespiratory events, MII/pH

## Abstract

Background: Feeding intolerance is a frequent diagnosis in very preterm infants. As seen in the FortiLat trial, human milk fortification with the new donkey milk-derived human milk fortifier (DF) seems to improve feeding tolerance in these infants. The aim of this study was to evaluate the effects of using the DF compared with bovine milk-derived fortifier (BF) on gastroesophageal reflux (GER) in very low birth weight (VLBW) infants. Methods: Over a total of 156 preterm infants were enrolled into the FortiLat trial (GA <32 weeks and birth weight <1500 g) and randomized into the BF arm or DF arm, and we selected all infants with clinical signs of GER and cardiorespiratory (CR) symptoms. All the infants underwent CR and multichannel intraluminal impedance and pH (MII/pH) monitoring associated with gastric ultrasound to evaluate GER and gastric emptying time. Results: 10 infants were enrolled, and 5 were in the DF arm. At MII/pH, infants enrolled into the DF arm showed a lower GER frequency than BF arm infants (*p* = 0.036). Half gastric emptying time was similar in DF and BF arm infants (*p* = 0.744). Conclusion: The use of donkey-derived human milk fortifier reduced the GER frequency and consequently should be recommended in infants with feeding intolerance.

## 1. Introduction

Very preterm and very low birth weight (VLBW) infants frequently experience feeding intolerance (FI) in the first weeks of life. FI is related to gastrointestinal anatomical and functional immaturity and decreased intestinal motility [1,2], and it is variably defined as the presence of emesis, visible bowel loops, increased abdominal girth and distension, presence of an abnormal gastric residual, and gastro-esophageal reflux (GER) [3]. GER is a physiological condition in preterm infants, and as FI is related to immaturity of the gastrointestinal tract, it is expressed through lower esophageal sphincter incontinence. The presence of FI symptoms such as increased gastric residual and abdominal distension, together with lower esophageal sphincter immaturity, can increase the frequency and worsen the characteristics of GER. Among symptoms of FI, regurgitation and vomiting are also characteristic signs of GER. However, in about 5% of cases, infants manifest cardiorespiratory (CR) events (apnea, bradycardia, blood oxygen desaturation) as atypical signs of GER [4,5,6]. In this population a significant association between GER and CR events has been recently demonstrated in 11% of cases [7]. Even if the temporal and causal association is still controversial, recent studies have demonstrated that in some patients with particular GER characteristics, there is a significant and causal association between these two events. The GER events involved in these associations are mainly weakly acidic suggesting that the empirical treatment with antacids is, in most of these cases, inappropriate [4,7].

The proper approach to the problem of GER in preterm infants should be aimed at improving feeding tolerance by adopting techniques and behaviors involving nutrition strategies (thickening of feeds, positioning, slow feeding) and able to reduce the frequency of refluxes. Among these strategies, the choice of the most proper nutrition can also influence GER. The use of breast or human milk is recommended, but in preterm infants there is the need to supplement it with additional nutrients to meet their nutritional requirements [8,9,10,11]. Most of these supplements are fortifiers derived from bovine milk, but concerns are arising as cow milk protein intake in the first month of life seems to be associated with allergies and intestinal inflammation in preterms [12,13]. Aceti et al. [14] demonstrated that human milk fortification based on a bovine milk-derived fortifier influenced the reflux index and weakly acidic reflux frequency. Based on the consideration that donkey milk has been suggested as a valid alternative for children allergic to cow’s milk proteins, due to its biochemical similarity to human milk, we hypothesized that this could be a suitable ingredient for developing an innovative human milk fortifier. A new donkey milk-derived human milk fortifier (DF) was developed as an alternative to bovine milk-derived human milk fortifiers (BF). We tested it through an RCT performed in our Center (FortiLat trial). This study suggests that DF improves feeding tolerance when compared with standard BF showing a lower number of failures (necrotizing enterocolitis, FI, death) and lower risk of FI episodes [15]. The evaluation of GER and gastric emptying, as a marker of FI, was evaluated by multichannel intraluminal impedance and pH monitoring (MII/pH) and gastric ultrasound (US) as secondary endpoints of the FortiLat trial [15,16].

The aim of this study is to evaluate the effects of the DF on VLBW and very preterm infants enrolled in the FortiLat trial with symptoms of GER and CR events.

## 2. Materials and Methods 

### 2.1. Study Design

All 156 infants included in the FortiLat trial (gestational age <32 weeks and birthweight ≤1500 g, exclusively fed with human milk) were considered for the present study.

After informed written parental consent was obtained, infants were randomized 1:1 by a software-generated list in one of the following groups: the control group (BF arm) underwent adjustable fortification with fortifier derived from bovine milk; the FortiLat group (DF arm) underwent adjustable fortification with fortifier derived from donkey milk. All patients were fed exclusively with human milk (mother’s own milk and/or donor breast milk) throughout the study. The different compositions of the two types of fortifiers can be observed in Table 1.

All infants were fed according to a regimen of Adjustable Fortification, based on BUN determination and started when reaching an enteral feeding ≥80 mL/kg/day. In the BF arm, Adjustable Fortification was conducted with a commercial multi-component fortifier (FM85 Nestlé) and a protein concentrate (Protifar Nutricia), derived from bovine milk, while in the DF arm it was conducted with a multi-component fortifier and a protein concentrate derived from donkey milk (FortiLat), not commercially available, and prepared according to current EU legislation on food for special medical purpose. 

Advancing of enteral feeds was strictly regulated according to the feeding protocol adopted in our NICU, based on the evaluation of signs of feeding intolerance. 

The detailed protocol of the FortiLat trial, the eligibility criteria, sample size calculation, settings, and randomization are specified in the FortiLat trial [15,16]. The study followed the Consolidate Standards of Reporting Trials. The neonates affected by severe gastrointestinal pathologies (necrotizing enterocolitis, colostomy, intestinal obstruction, symptoms of peritonitis, presence of blood in the feces), chromosomal abnormalities or major malformations, hereditary metabolic diseases, intravascular disseminated coagulopathy (IDC), shock, patent ductus arteriosus (PDA) requiring medical care or surgery at time of randomization, and severe renal failure (serum creatinine >2 mg/dL) were excluded.

At day 21 since beginning of fortification, infants showing typical signs of GER (excessive regurgitations or vomiting >3/day any amount or > once/day half the feed, and belching) and CR symptoms (apneas/desaturations/bradycardia, paleness, cyanosis) >3/day were enrolled into the present study. Enrolled infants underwent synchronized multichannel intraluminal impedance/pH (MII/pH) and CR monitoring and then gastric emptying US assessment as specified below. Day 21 was arbitrarily chosen for the evaluation of GER, considering 3 weeks of fortification to be the minimum effective time to evaluate the differences caused by the different fortifiers.

### 2.2. MII/pH Monitoring

The evaluation of GER events was performed through MII/pH monitoring (Sleuth monitoring system). The recommendations for the procedure and the identification of GER events have already been described in a previous work by our group [17]. A MII-GER event was defined as a retrograde drop of impedance to 50% of the basal value for at least 5 s, starting in the most distal channel, proceeding to one or more proximal channels and followed by a recovery of the impedance baseline values. A reflux reaching the two most proximal channels is defined as proximal GER; pH-GER events, defined as drop of pH value below 4 longer than 5 s and not associated with a MII-GER event, were considered in the analysis as well.

The following features were evaluated:

- MII-GER frequency, expressed as reflux events/h;
- Bolus reflux extent (BRE): the proximal extent reached by the refluxate, indicated by the number of channels sequentially involved, expressed as number of channels;
- Bolus clearance time (BCT): duration of a reflux from the drop to 50% of the impedance baseline value to its recovery recorded in the distal impedance channel, expressed as seconds;
- Bolus exposure index (BEI): total percentage of time bolus reflux was detected by multichannel intraluminal impedance, expressed as a percentage;
- pH-GER frequency, expressed as reflux events/h;

Reflux index (RI): total percentage of time with pH < 4, expressed as a percentage. Each MII-GER event was defined as weakly acidic (4 ≤ pH > 7), acidic (pH < 4), or weakly alkaline (pH ≥ 7) according to the minimum pH value reached during each event [17,18,19].

### 2.3. CR Monitoring

The VitaGuard VG3100 system (Getemed Medizin und Information stechnik AG, Teltow, Germany), equipped with Signal Extraction Technology (Masimo Corp. Irvine, CA, USA), was used to monitor CR. Heart rate, transcutaneous blood oxygen saturation, and respiratory rate were measured by a pulse-oximetry sensor placed on the right wrist or foot and three cardiac electrodes placed on the chest. CR tracings were “visually” analyzed by a trained operator blinded to the MII/pH tracings using VitaWin3^®^ evaluation software 3.1.

CR events were defined as episodes of apnea lasting more than 20 s or over 5 s if followed by desaturation or bradycardia, episodes of desaturation with blood oxygen saturation below 80%, and episodes of bradycardia with heart rate below 80 beats per minute [7,20]. Minimum duration of bradycardia and blood oxygen desaturation events to be considered for the analysis was 4 s.

### 2.4. Synchronized MII/pH and CR Monitoring

CR and MII/pH monitors were synchronized using an external reference (i.e., Internet time) and digitally marking each tracing at the beginning of the study. The specific times of each CR event were adjusted for the offset between the clocks of the CR and MII/pH monitors. The symptom association probability (SAP) index was calculated to evaluate temporal associations between GER and CR events [21]. SAP index value >95% identified patients with a significant number of GER–CR associations.

The primary outcome of the study was the MII-GER frequency, and secondary outcomes were reflux characteristics, half gastric emptying time (T/2), and the SAP index.

### 2.5. Gastric US

Gastric antral transit was used as a proxy of gastric emptying. This was determined by measuring ultrasonically the changes in the antral cross-sectional area (ACSA) that occur after a feed [22]. Serial measurements of ACSA were made before, during, and after administration of feeds every 10 min for 90 min. All gastric US scans were performed and read by a single trained operator, who was blind to the type of fortifier used.

The gastric emptying curve was calculated from the ultrasound series and represented by the best fitting polynomial function (*R*^2^ > 0.98. *p* < 0.001) of the ACSA values. On this basis, the half gastric emptying time (T/2), calculated as the time required to achieve a 50% reduction in ACSA values, was obtained [22]. Half gastric emptying time was evaluated as a secondary outcome.

### 2.6. Statistical Analysis

Statistical analyses were performed using the STATISTICA software package for Windows (StatSoft, Inc., Tulsa, Oklahoma, USA). Results are expressed as median and interquartile range (IQR) if not otherwise specified, and the p value was set at 0.05. The distribution of the variables was evaluated by the Shapiro–Wilk test. Student’s *t*-test was used to evaluate differences between normally distributed variables, and the Mann–Whitney test for unpaired data was used for non-normally distributed variables.

### 2.7. Ethics

This is an ancillary study from the FortiLat trial [15,16] and was performed in the NICU of Turin University. FortiLat trial was approved by Ethics Committee (AN: 0025847. 27/05/2014) and registered (http://www.isrctn.com/ISRCTN70022881). Recruitment period was 27 November 2014 to 22 December 2016. Written informed consent was obtained from the parents of all included newborns before enrollment. 

## 3. Results

**Figure 1:**
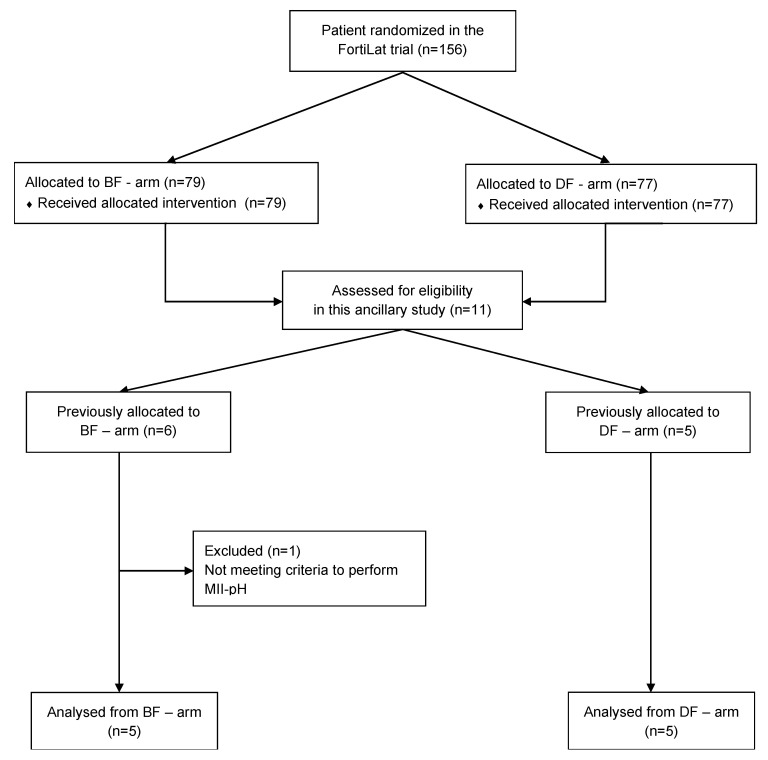
Diagram of patient allocation. BF arm: arm treated with bovine milk-derived human milk fortifier; DF arm: arm treated with donkey milk-derived human milk fortifier; MII-pH: multichannel intraluminal impedance and pH monitoring.

Out of 156 newborns enrolled in the FortiLat trial [15], 11 (7.05%) met the inclusion criteria. Six were allocated in the BF arm and 5 in the DF arm. One infant, allocated in the BF arm, was excluded because his weight (1120 g) at 21 days of fortification was considered too low to perform MII/pH safely. A total of 10 (6.4%) infants were enrolled and underwent synchronized MII/pH and CR monitoring in order to evaluate GER and gastric US in order to evaluate gastric emptying. A flow chart is available in Figure 1.

These 10 infants included in the analysis had a mean ± SD gestational age of 30 ± 1.3 weeks and birth weight of 1215 ± 424 g. No adverse effects associated with MII/pH and gastric US have been reported in any patients enrolled.

The anthropometric characteristics of infants included in this study are summarized in Table 2. No significant differences were found between the two groups for any of the variables considered.

Overall, MII showed a median MII-GER frequency per hour of 3.05 (2.22–4.43) with a BCT of 19.29 (16.29–23.36) s, a BRE of 3.42 (3.26–3.71) cm, and a BEI of 1.46 (1.18–3.04) %. The median frequency of MII-GER was 2.47 (1.50–4.04), 0.50 (0.14–0.99), and 0.08 (0.00–0.21) GER/h for weakly acidic, acid, and weakly alkaline GER respectively. pH-metry showed a median GER frequency per hour of 1.49 (0.61–2.14). The median half gastric emptying time was 46.02 (43.24–48.72) min.

DF arm infants had a significant lower frequency of MII-GER with a median of 2.02 (1.95–3.26) versus 4.82 (2.84–5.94) in BF arm infants (*p* = 0.036). Detailed MII/pH and gastric emptying data are reported in Table 3. We found a total of 73 CR events, 6 (2–14) events per patient: 7 (9.59%) apneas >20 s, 12 (16.44%) apneas <20 s associated with desaturations and/or bradycardias, 49 (67.12%) isolated desaturations, and 5 (6.58%) isolated bradycardias. The median value for blood oxygen desaturation events was 75 (71–78) %, the lowest was 54%. Median value for bradycardia was 67 (63–78) beats per minute with a lowest of 58 beats per minute. The median duration of apnea events was 12 (8–22) s, and the longest apnea recorded was 38 s. Detailed CR monitoring data are reported in Table 3.

No infant had a significant association between GER-CR events (SAP = 0.86 ± 0.07), and there were no differences in SAP between DF arm and BF arm infants.

The post-hoc analysis based on the obtained sample size, considering an α values of 0.5, revealed a power of 80.6% referring to the primary outcome (MII-GER frequency) values observed in the two arms.

## 4. Discussion

This research represents an ancillary study of FortiLat trial [15,16], which involved infants born at gestational age <32 weeks and birthweight ≤1500 g, exclusively fed with fortified human milk, randomly assigned to DF arm or BF arm. In the present study, we evaluated by “state-of-the-art” techniques a research topic not addressed in the FortiLat trial main paper: the effect of donkey milk-derived human milk fortifier on gastroesophageal reflux in infants with typical GER symptoms and cardiorespiratory events. The main outcome of the study was the reflux frequency, and we found a significant reduction of the frequency of all MII-GER and of weakly acidic MII-GER in the DF arm compared to BF arm infants.

In preterm infants, the incidence of the diagnosis of GER based on symptoms is highly variable in literature and ranges between 2 and 26% [23]. In our neonatal intensive care unit, we found that the burden of preterm infants with GER symptoms was 11%, and almost 38% of them showed respiratory symptoms associated with GER typical symptoms [17]. Thus, we expected to find a population with typical GER symptoms, and cardiorespiratory showed symptoms of approximately 6.5%. According to these data, of the 156 enrolled in the FortiLat trial we found 10 (6.4%) infants eligible for this study. Although the sample studied was very small, a post-hoc analysis showed that the study achieved a power of 80% considering our main outcome.

Since the two fortifiers are isoproteic and isocaloric, we speculate that the quality of donkey milk protein could be responsible of this result.

The lower frequency of refluxes in the DF group mainly affected the weakly acidic ones, which are the most frequent in infants and could be symptomatic [17]. On the contrary, the incidence of acid refluxes in this arm was higher. The RI evaluated with pH-metry, although not pathological, was higher in the DF arm infants. We speculate this is probably due to the lower buffering capacity of DF compared to BF (Table 1). We previously reported that the low molecular weight of the proteins contained in hydrolyzed milk formula produced an increased buffer capacity with respect to the same not-hydrolyzed milk formula [24]. The lower buffering capacity of DF could be explained by the different composition of the two fortifiers since DF is composed by protein macromolecules while BF by free amino acids. It is remarkable that the two fortifiers contain very similar amounts of glycides while having different compositions: DF contains mostly lactose, while BF contains mostly maltodextrin. Currently, there are no studies evaluating the effects of these two components on GER in premature infants; however, the composition of DF is more similar to that of breast milk, which is known to be protective against GER [25].

Previous studies demonstrated an increased risk of necrotizing enterocolitis due to the neutralization of the antimicrobial effect of the physiological low gastric pH in preterm infants treated with antacid [26]. In this context, the lower buffering effects on gastric pH of DF could be a protective factor, prevent infections and necrotizing enterocolitis, and should be studied in further larger prospective trials. Despite the expectations, we failed to detect any differences in gastric emptying time between the two arms. However, it should be considered that the method used was characterized by a great variability between the infants evaluated, and a much larger sample should be studied to detect significant differences between the two groups.

A limitation of the study is due to the characteristics of the available MII/pH probes that are not suitable for infants under 1200 g at the time of MII/pH; therefore, our results cannot be generalized to infants below this weight limit.

Finally, we have evaluated GER–CR temporal associations. GER and CR events are very frequent in preterm infants, but patients with a significant temporal association between these events are very rare. The execution of the MII/pH synchronized with CR monitoring allowed us to exclude this association in all the subjects studied, avoiding unnecessary and potentially harmful therapies.

It is known that GER is one of the signs of FI, and our data are in line with the FortiLat trial results, suggesting a favorable effect of the donkey milk fortifier on it.

## 5. Conclusions 

In this study we compared the effects on GER of a new donkey milk fortifier (DF) to a cow’s milk fortifier in a group of VLBW infants fed exclusively with human milk with typical GER symptoms and cardiorespiratory symptoms. Our data showed a reduction in the frequency of GER in infants of the DF arm. Considering that the two fortifiers are isoproteic and isocaloric, we speculate that the quality of donkey components is responsible for the better tolerance found in the DF arm. If these results will be confirmed by larger studies, DF could be recommended in VLBW infants with GER symptoms.

## Figures and Tables

**Table 1:** Composition of BF and DF; values per 100 g of product.

	BF	DF
Protein g (Nx6.25)	20.0	22.5
Carbohydrate (g) Of which:	66.0	59.0
Lactose (g)	6.0	59.0
Maltodextrin (g)	60.0	0.0
Fat (g)	0.4	3.6
Kcal	385	390
Kcal/g protein	18.8	15.6
Calcium (mg)	1500	938
Phosphate (mg)	900	734
Osmolality (mosm/kg)	453	441
pH	6.8	6.7
Buffering capacity (mmol l-1/dph)	12.2	9.2

BF: bovine milk-derived human milk fortifier. DF: donkey milk-derived human milk fortifier.

**Table 2:** Anthropometric characteristics per arm at enrollment, expressed in mean ± sd.

	BF arm	DF arm
Cases (no.)	5 (50%)	5 (50%)
Gender (male)	3 (60%)	2 (40%)
Gestational age (weeks)	30.0 ± 0.7	30.2 ± 1.8
Birth weight (g)	1398 ± 469	1032 ± 320.2
Birth length (cm)	37.9 ± 4.8	36.9 ± 1.4
Birth head circumference (cm)	27.9 ± 2.5	26.8 ± 2.1
Age (days)	35.2 ± 6.8	39.6 ± 9.1
Post menstrual age (weeks)	35 ± 0.9	36 ± 2.3
Weight (g)	1908 ± 449	1644 ± 386
Length (cm)	42.6 ± 4.3	40.56 ± 3.6
Head circumference (cm)	29.52 ± 2.5	29.5 ± 1.9

BF arm: arm treated with bovine milk-derived human milk fortifier. DF arm: arm treated with donkey milk-derived human milk fortifier.

**Table 3:** MII/pH, gastric US, and CR monitoring data. Comparison between BF arm and DF arm.

			BF	DF	*p*
		Cases (no.)	5 (50%)	5 (50%)	-
MII-GER	MII-GER	GER Frequency (GER/h)	4.82 (2.84–5.94)	2.02 (1.95–3.26)	0.036
		proximal GER Frequency (GER/h)	1.97 (0.94–2.82)	0.95 (0.79–1.42)	0.246
		BCT (s)	17.91 (16.45–24.14)	20.66 (16.24–21.01)	0.895
		BRE (cm)	3.44 (3.20–3.71)	3.41 (3.26–3.71)	0.705
		BEI (%)	3.41 (1.28–3.73)	1.44 (0.96–1.47)	0.064
	MII-WA-GER	GER Frequency (GER/h)	4.50 (2.68–4.82)	1.43 (1.35–2.47)	0.024
		proximal GER Frequency (GER/h)	1.89 (0.94–2.10)	0.48 (0.43–1.26)	0.156
		BCT (s)	18.90 (16.70–25.04)	20.37 (19.77–21.64)	0.774
		BRE (cm)	3.37 (3.21–3.93)	3.40 (3.24–3.91)	0.865
	MII-ACID-GER	GER Frequency (GER/h)	0.08 (0.05–1.13)	0.53 (0.47–0.78)	0.611
		proximal GER Frequency (GER/h)	0.06 (0.00–0.08)	0.32 (0.11–0.48)	0.435
		BCT (s)	18.60 (16.70–20.30)	21.88 (11.25–23.62)	0.825
		BRE (cm)	3.37 (3.00–3.73)	3.33 (3.33–4.00)	0.335
	MII-WALK-GER	GER Frequency (GER/h)	0.13 (0.00–0.24)	0.06 (0.00–0.11)	0.359
		proximal GER Frequency (GER/h)	0.00 (0.00–0.00)	0.00 (0.00–0.05)	0.397
		BCT (s)	11.40 (0.00–14.10)	8.70 (0.00–10.32)	0.981
		BRE (cm)	2.33 (0.00–2.67)	2.00 (0.00–2.67)	0.895
pH-GER	pH-GER	GER Frequency (GER/h)	0.55 (0.05–0.77)	1.89 (1.79–2.22)	0.122
		RI (%)	1.29 (0.04–2.23)	3.89 (3.60–6.35)	0.388
Gastric US	Gastric emptying time	T/2 (min)	45.03 (42.74–47.02)	48.57 (44.73–48.77)	0.744
CR-monitor	CR events	Apnea (no.)	4	3	-
		Apnea + Desaturation +/− Bradycardia	6	6	-
		Desaturation	28	21	-
		Bradycardia	2	3	-

BF: bovine milk-derived human milk fortifier; DF: donkey milk-derived human milk fortifier; MII: multichannel intraluminal impedance; GER: gastroesophageal reflux; BCT: bolus clearance time; BRE: bolus reflux extent; BEI: bolus exposure index; WA: weakly acidic; WALK: weakly alkaline; RI: reflux index; US: ultrasound; CR: cardiorespiratory.

## Abbreviations

VLBW	Very Low Birth Weight
FI	Feeding Intolerance
GER	Gastroesophageal Reflux
CR	Cardiorespiratory
DF	Donkey Fortifier
BF	Bovine Fortifier
MII/pH	Multichannel Intraluminal Impedance and pH Monitoring
US	Ultrasound
BRE	Bolus Reflux Extent
BCT	Bolus Clearance Time
BEI	Bolus Exposure Index
RI	Reflux Index
SAP	Symptom Association Probability
ACSA	Antral Cross-Sectional Area
